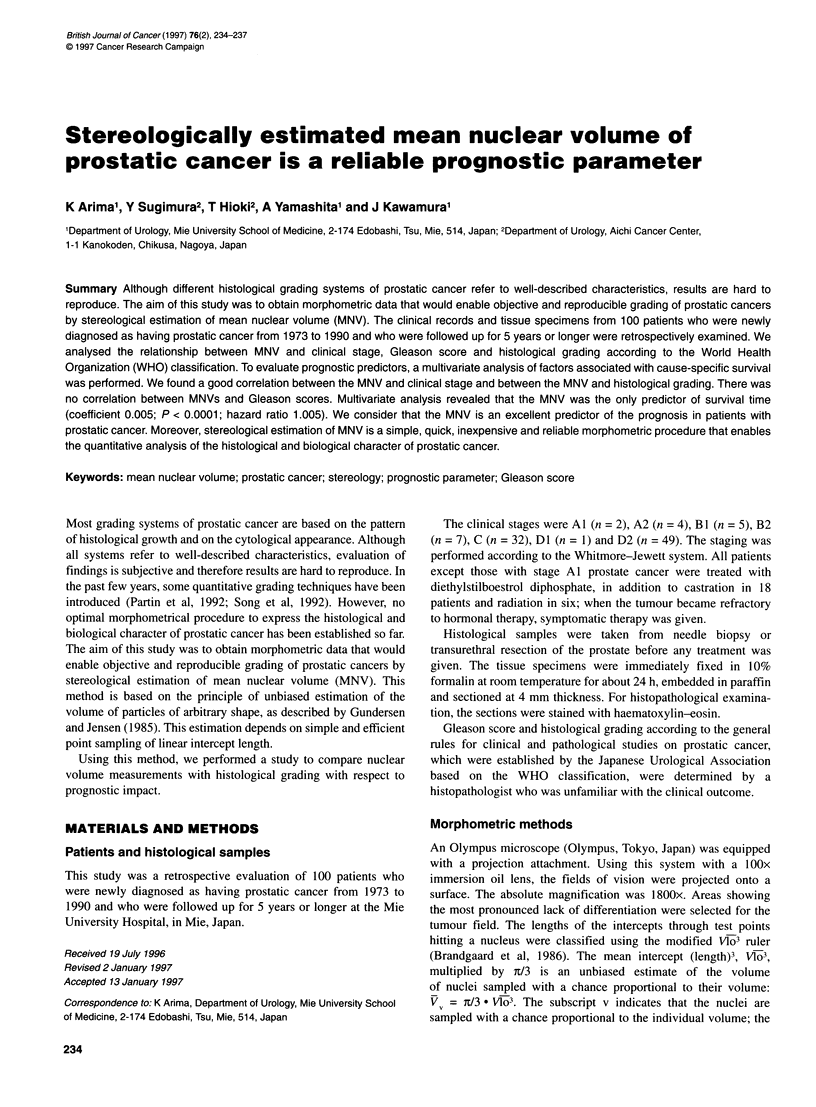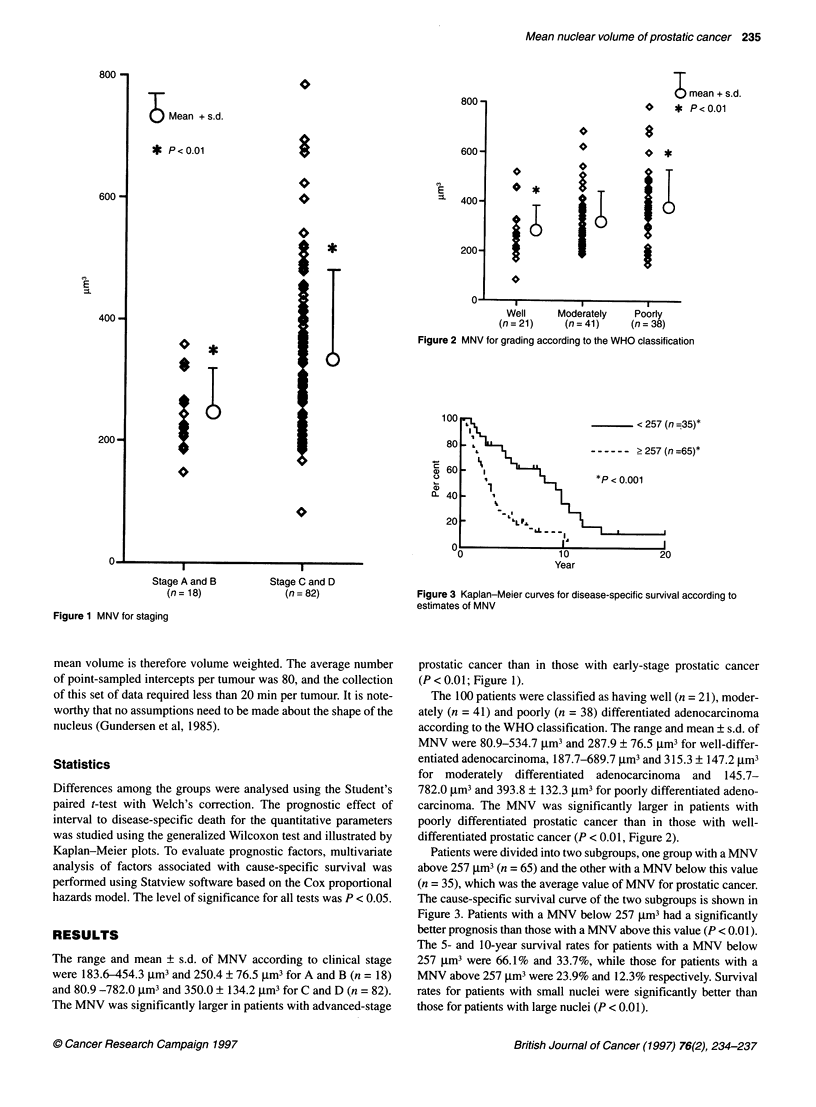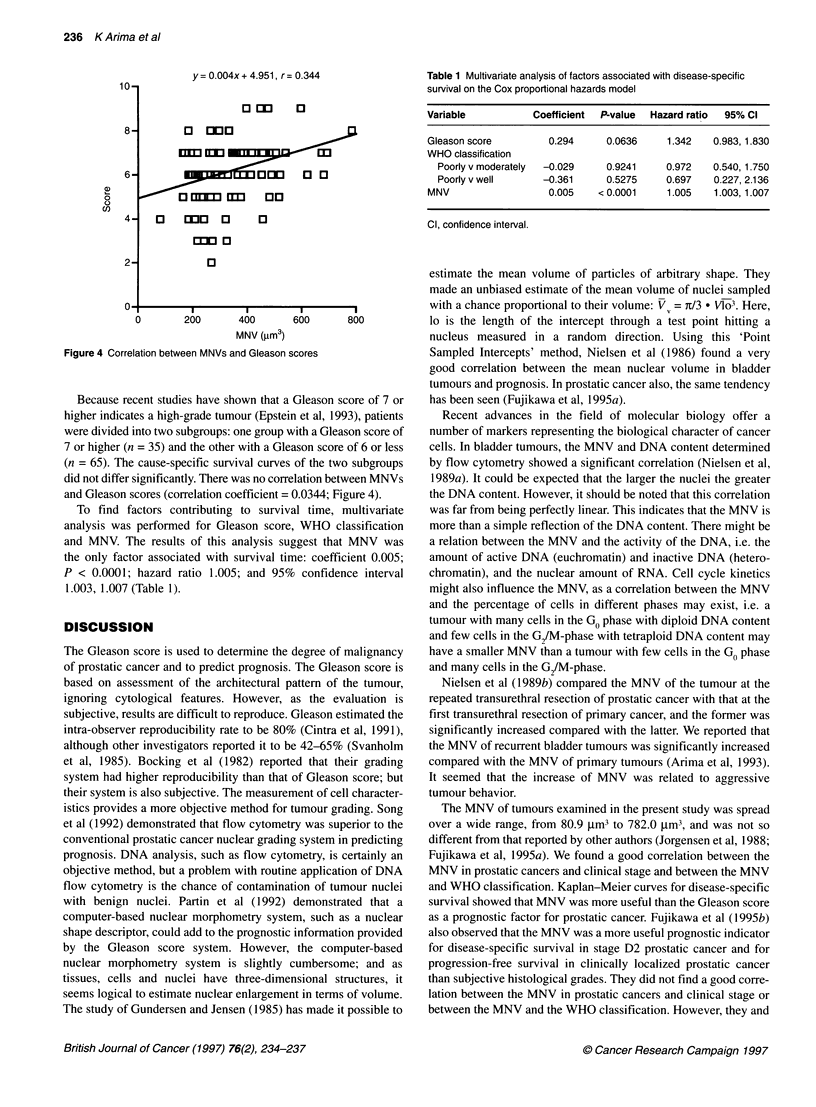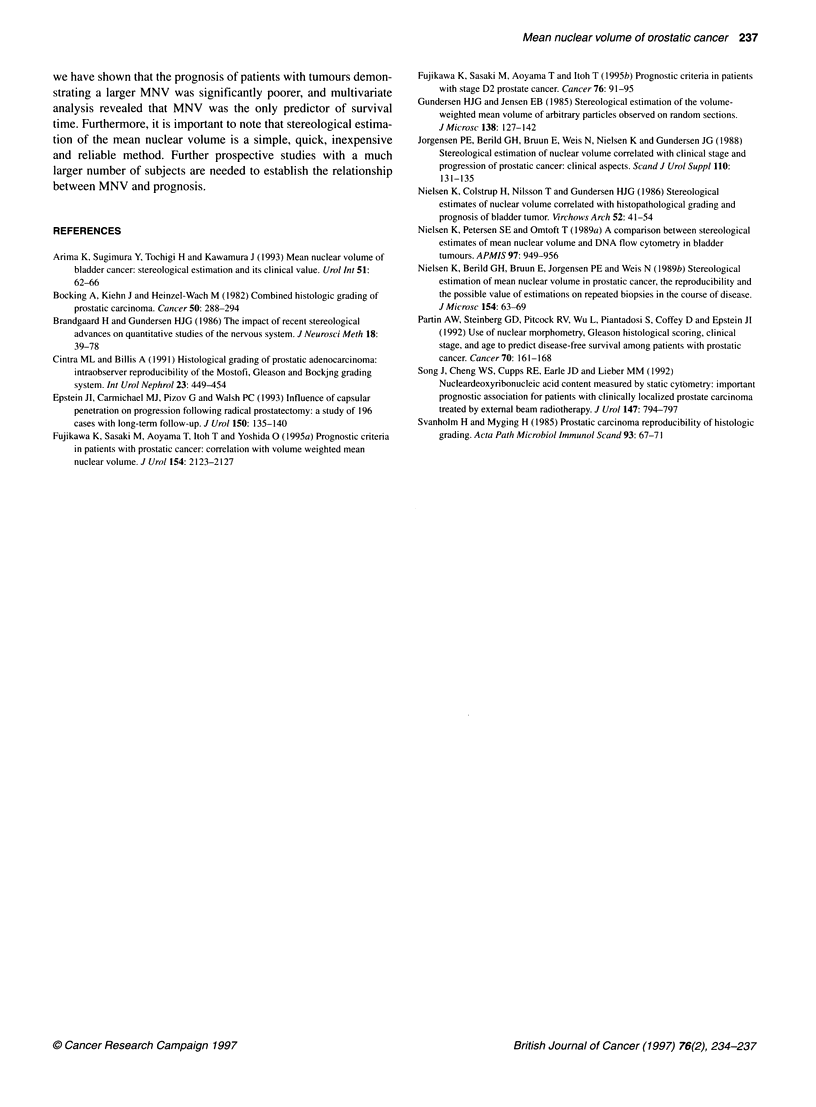# Stereologically estimated mean nuclear volume of prostatic cancer is a reliable prognostic parameter.

**DOI:** 10.1038/bjc.1997.367

**Published:** 1997

**Authors:** K. Arima, Y. Sugimura, T. Hioki, A. Yamashita, J. Kawamura

**Affiliations:** Department of Urology, Mie University School of Medicine, Tsu, Japan.

## Abstract

Although different histological grading systems of prostatic cancer refer to well-described characteristics, results are hard to reproduce. The aim of this study was to obtain morphometric data that would enable objective and reproducible grading of prostatic cancers by stereological estimation of mean nuclear volume (MNV). The clinical records and tissue specimens from 100 patients who were newly diagnosed as having prostatic cancer from 1973 to 1990 and who were followed up for 5 years or longer were retrospectively examined. We analysed the relationship between MNV and clinical stage, Gleason score and histological grading according to the World Health Organization (WHO) classification. To evaluate prognostic predictors, a multivariate analysis of factors associated with cause-specific survival was performed. We found a good correlation between the MNV and clinical stage and between the MNV and histological grading. There was no correlation between MNVs and Gleason scores. Multivariate analysis revealed that the MNV was the only predictor of survival time (coefficient 0.005; P < 0.0001; hazard ratio 1.005). We consider that the MNV is an excellent predictor of the prognosis in patients with prostatic cancer. Moreover, stereological estimation of MNV is a simple, quick, inexpensive and reliable morphometric procedure that enables the quantitative analysis of the histological and biological character of prostatic cancer.


British Journal of Cancer (1997) 76(2), 234–237
© 1997 Cancer Research Campaign

### Stereologically estimated mean nuclear volume of prostatic cancer is a reliable prognostic parameter

**K Arima[1], Y Sugimura[2], T Hioki[2], A Yamashita[1] and J Kawamura[1]**

[1]Department of Urology, Mie University School of Medicine, 2-174 Edobashi, Tsu, Mie, 514, Japan; [2]Department of Urology, Aichi Cancer Center, 1-1 Kanokoden, Chikusa, Nagoya, Japan

**Summary** Although different histological grading systems of prostatic cancer refer to well-described characteristics, results are hard to reproduce. The aim of this study was to obtain morphometric data that would enable objective and reproducible grading of prostatic cancers by stereological estimation of mean nuclear volume (MNV). The clinical records and tissue specimens from 100 patients who were newly diagnosed as having prostatic cancer from 1973 to 1990 and who were followed up for 5 years or longer were retrospectively examined. We analysed the relationship between MNV and clinical stage, Gleason score and histological grading according to the World Health Organization (WHO) classification. To evaluate prognostic predictors, a multivariate analysis of factors associated with cause-specific survival was performed. We found a good correlation between the MNV and clinical stage and between the MNV and histological grading. There was no correlation between MNVs and Gleason scores. Multivariate analysis revealed that the MNV was the only predictor of survival time (coefficient 0.005; $P < 0.0001$; hazard ratio 1.005). We consider that the MNV is an excellent predictor of the prognosis in patients with prostatic cancer. Moreover, stereological estimation of MNV is a simple, quick, inexpensive and reliable morphometric procedure that enables the quantitative analysis of the histological and biological character of prostatic cancer.

**Keywords:** mean nuclear volume; prostatic cancer; stereology; prognostic parameter; Gleason score

Most grading systems of prostatic cancer are based on the pattern of histological growth and on the cytological appearance. Although all systems refer to well-described characteristics, evaluation of findings is subjective and therefore results are hard to reproduce. In the past few years, some quantitative grading techniques have been introduced (Partin et al, 1992; Song et al, 1992). However, no optimal morphometrical procedure to express the histological and biological character of prostatic cancer has been established so far. The aim of this study was to obtain morphometric data that would enable objective and reproducible grading of prostatic cancers by stereological estimation of mean nuclear volume (MNV). This method is based on the principle of unbiased estimation of the volume of particles of arbitrary shape, as described by Gundersen and Jensen (1985). This estimation depends on simple and efficient point sampling of linear intercept length.

Using this method, we performed a study to compare nuclear volume measurements with histological grading with respect to prognostic impact.

### MATERIALS AND METHODS

### Patients and histological samples

This study was a retrospective evaluation of 100 patients who were newly diagnosed as having prostatic cancer from 1973 to 1990 and who were followed up for 5 years or longer at the Mie University Hospital, in Mie, Japan.

*Received 19 July 1996*
*Revised 2 January 1997*
*Accepted 13 January 1997*

*Correspondence to:* K Arima, Department of Urology, Mie University School of Medicine, 2-174 Edobashi, Tsu, Mie, 514, Japan

The clinical stages were A1 ($n$ = 2), A2 ($n$ = 4), B1 ($n$ = 5), B2 ($n$ = 7), C ($n$ = 32), D1 ($n$ = 1) and D2 ($n$ = 49). The staging was performed according to the Whitmore–Jewett system. All patients except those with stage A1 prostate cancer were treated with diethylstilboestrol diphosphate, in addition to castration in 18 patients and radiation in six; when the tumour became refractory to hormonal therapy, symptomatic therapy was given.

Histological samples were taken from needle biopsy or transurethral resection of the prostate before any treatment was given. The tissue specimens were immediately fixed in 10% formalin at room temperature for about 24 h, embedded in paraffin and sectioned at 4 mm thickness. For histopathological examination, the sections were stained with haematoxylin–eosin.

Gleason score and histological grading according to the general rules for clinical and pathological studies on prostatic cancer, which were established by the Japanese Urological Association based on the WHO classification, were determined by a histopathologist who was unfamiliar with the clinical outcome.

### Morphometric methods

An Olympus microscope (Olympus, Tokyo, Japan) was equipped with a projection attachment. Using this system with a 100× immersion oil lens, the fields of vision were projected onto a surface. The absolute magnification was 1800×. Areas showing the most pronounced lack of differentiation were selected for the tumour field. The lengths of the intercepts through test points hitting a nucleus were classified using the modified $\overline{Vlo^3}$ ruler (Brandgaard et al, 1986). The mean intercept (length)$^3$, $\overline{Vlo^3}$, multiplied by $\pi/3$ is an unbiased estimate of the volume of nuclei sampled with a chance proportional to their volume: $\bar{V}_v = \pi/3 \cdot \overline{Vlo^3}$. The subscript v indicates that the nuclei are sampled with a chance proportional to the individual volume; the

Figure 1 MNV for staging

Figure 2 MNV for grading according to the WHO classification

Figure 3 Kaplan–Meier curves for disease-specific survival according to estimates of MNV

mean volume is therefore volume weighted. The average number of point-sampled intercepts per tumour was 80, and the collection of this set of data required less than 20 min per tumour. It is noteworthy that no assumptions need to be made about the shape of the nucleus (Gundersen et al, 1985).

### Statistics

Differences among the groups were analysed using the Student's paired $t$-test with Welch's correction. The prognostic effect of interval to disease-specific death for the quantitative parameters was studied using the generalized Wilcoxon test and illustrated by Kaplan–Meier plots. To evaluate prognostic factors, multivariate analysis of factors associated with cause-specific survival was performed using Statview software based on the Cox proportional hazards model. The level of significance for all tests was $P < 0.05$.

### RESULTS

The range and mean ± s.d. of MNV according to clinical stage were 183.6–454.3 μm³ and 250.4 ± 76.5 μm³ for A and B ($n = 18$) and 80.9 –782.0 μm³ and 350.0 ± 134.2 μm³ for C and D ($n = 82$). The MNV was significantly larger in patients with advanced-stage prostatic cancer than in those with early-stage prostatic cancer ($P < 0.01$; Figure 1).

The 100 patients were classified as having well ($n = 21$), moderately ($n = 41$) and poorly ($n = 38$) differentiated adenocarcinoma according to the WHO classification. The range and mean ± s.d. of MNV were 80.9–534.7 μm³ and 287.9 ± 76.5 μm³ for well-differentiated adenocarcinoma, 187.7–689.7 μm³ and 315.3 ± 147.2 μm³ for moderately differentiated adenocarcinoma and 145.7–782.0 μm³ and 393.8 ± 132.3 μm³ for poorly differentiated adenocarcinoma. The MNV was significantly larger in patients with poorly differentiated prostatic cancer than in those with well-differentiated prostatic cancer ($P < 0.01$, Figure 2).

Patients were divided into two subgroups, one group with a MNV above 257 μm³ ($n = 65$) and the other with a MNV below this value ($n = 35$), which was the average value of MNV for prostatic cancer. The cause-specific survival curve of the two subgroups is shown in Figure 3. Patients with a MNV below 257 μm³ had a significantly better prognosis than those with a MNV above this value ($P < 0.01$). The 5- and 10-year survival rates for patients with a MNV below 257 μm³ were 66.1% and 33.7%, while those for patients with a MNV above 257 μm³ were 23.9% and 12.3% respectively. Survival rates for patients with small nuclei were significantly better than those for patients with large nuclei ($P < 0.01$).

© Cancer Research Campaign 1997

**Figure 4** Correlation between MNVs and Gleason scores

Because recent studies have shown that a Gleason score of 7 or higher indicates a high-grade tumour (Epstein et al, 1993), patients were divided into two subgroups: one group with a Gleason score of 7 or higher ($n$ = 35) and the other with a Gleason score of 6 or less ($n$ = 65). The cause-specific survival curves of the two subgroups did not differ significantly. There was no correlation between MNVs and Gleason scores (correlation coefficient = 0.0344; Figure 4).

To find factors contributing to survival time, multivariate analysis was performed for Gleason score, WHO classification and MNV. The results of this analysis suggest that MNV was the only factor associated with survival time: coefficient 0.005; $P$ < 0.0001; hazard ratio 1.005; and 95% confidence interval 1.003, 1.007 (Table 1).

### DISCUSSION

The Gleason score is used to determine the degree of malignancy of prostatic cancer and to predict prognosis. The Gleason score is based on assessment of the architectural pattern of the tumour, ignoring cytological features. However, as the evaluation is subjective, results are difficult to reproduce. Gleason estimated the intra-observer reproducibility rate to be 80% (Cintra et al, 1991), although other investigators reported it to be 42–65% (Svanholm et al, 1985). Bocking et al (1982) reported that their grading system had higher reproducibility than that of Gleason score; but their system is also subjective. The measurement of cell characteristics provides a more objective method for tumour grading. Song et al (1992) demonstrated that flow cytometry was superior to the conventional prostatic cancer nuclear grading system in predicting prognosis. DNA analysis, such as flow cytometry, is certainly an objective method, but a problem with routine application of DNA flow cytometry is the chance of contamination of tumour nuclei with benign nuclei. Partin et al (1992) demonstrated that a computer-based nuclear morphometry system, such as a nuclear shape descriptor, could add to the prognostic information provided by the Gleason score system. However, the computer-based nuclear morphometry system is slightly cumbersome; and as tissues, cells and nuclei have three-dimensional structures, it seems logical to estimate nuclear enlargement in terms of volume. The study of Gundersen and Jensen (1985) has made it possible to

**Table 1** Multivariate analysis of factors associated with disease-specific survival on the Cox proportional hazards model

| Variable | Coefficient | *P*-value | Hazard ratio | 95% CI |
|---|---|---|---|---|
| Gleason score | 0.294 | 0.0636 | 1.342 | 0.983, 1.830 |
| WHO classification | | | | |
| Poorly v moderately | –0.029 | 0.9241 | 0.972 | 0.540, 1.750 |
| Poorly v well | –0.361 | 0.5275 | 0.697 | 0.227, 2.136 |
| MNV | 0.005 | < 0.0001 | 1.005 | 1.003, 1.007 |

CI, confidence interval.

estimate the mean volume of particles of arbitrary shape. They made an unbiased estimate of the mean volume of nuclei sampled with a chance proportional to their volume: $\bar{V}_v = \pi/3 \bullet \overline{lo^3}$. Here, lo is the length of the intercept through a test point hitting a nucleus measured in a random direction. Using this 'Point Sampled Intercepts' method, Nielsen et al (1986) found a very good correlation between the mean nuclear volume in bladder tumours and prognosis. In prostatic cancer also, the same tendency has been seen (Fujikawa et al, 1995*a*).

Recent advances in the field of molecular biology offer a number of markers representing the biological character of cancer cells. In bladder tumours, the MNV and DNA content determined by flow cytometry showed a significant correlation (Nielsen et al, 1989*a*). It could be expected that the larger the nuclei the greater the DNA content. However, it should be noted that this correlation was far from being perfectly linear. This indicates that the MNV is more than a simple reflection of the DNA content. There might be a relation between the MNV and the activity of the DNA, i.e. the amount of active DNA (euchromatin) and inactive DNA (heterochromatin), and the nuclear amount of RNA. Cell cycle kinetics might also influence the MNV, as a correlation between the MNV and the percentage of cells in different phases may exist, i.e. a tumour with many cells in the $G_0$ phase with diploid DNA content and few cells in the $G_2$/M-phase with tetraploid DNA content may have a smaller MNV than a tumour with few cells in the $G_0$ phase and many cells in the $G_2$/M-phase.

Nielsen et al (1989*b*) compared the MNV of the tumour at the repeated transurethral resection of prostatic cancer with that at the first transurethral resection of primary cancer, and the former was significantly increased compared with the latter. We reported that the MNV of recurrent bladder tumours was significantly increased compared with the MNV of primary tumours (Arima et al, 1993). It seemed that the increase of MNV was related to aggressive tumour behavior.

The MNV of tumours examined in the present study was spread over a wide range, from 80.9 μm³ to 782.0 μm³, and was not so different from that reported by other authors (Jorgensen et al, 1988; Fujikawa et al, 1995*a*). We found a good correlation between the MNV in prostatic cancers and clinical stage and between the MNV and WHO classification. Kaplan–Meier curves for disease-specific survival showed that MNV was more useful than the Gleason score as a prognostic factor for prostatic cancer. Fujikawa et al (1995*b*) also observed that the MNV was a more useful prognostic indicator for disease-specific survival in stage D2 prostatic cancer and for progression-free survival in clinically localized prostatic cancer than subjective histological grades. They did not find a good correlation between the MNV in prostatic cancers and clinical stage or between the MNV and the WHO classification. However, they and

© Cancer Research Campaign 1997

we have shown that the prognosis of patients with tumours demonstrating a larger MNV was significantly poorer, and multivariate analysis revealed that MNV was the only predictor of survival time. Furthermore, it is important to note that stereological estimation of the mean nuclear volume is a simple, quick, inexpensive and reliable method. Further prospective studies with a much larger number of subjects are needed to establish the relationship between MNV and prognosis.

### REFERENCES

Arima K, Sugimura Y, Tochigi H and Kawamura J (1993) Mean nuclear volume of bladder cancer: stereological estimation and its clinical value. *Urol Int* **51**: 62–66

Bocking A, Kiehn J and Heinzel-Wach M (1982) Combined histologic grading of prostatic carcinoma. *Cancer* **50**: 288–294

Brandgaard H and Gundersen HJG (1986) The impact of recent stereological advances on quantitative studies of the nervous system. *J Neurosci Meth* **18**: 39–78

Cintra ML and Billis A (1991) Histological grading of prostatic adenocarcinoma: intraobserver reproducibility of the Mostofi, Gleason and Bockjng grading system. *Int Urol Nephrol* **23**: 449–454

Epstein JI, Carmichael MJ, Pizov G and Walsh PC (1993) Influence of capsular penetration on progression following radical prostatectomy: a study of 196 cases with long-term follow-up. *J Urol* **150**: 135–140

Fujikawa K, Sasaki M, Aoyama T, Itoh T and Yoshida O (1995*a*) Prognostic criteria in patients with prostatic cancer: correlation with volume weighted mean nuclear volume. *J Urol* **154**: 2123–2127

Fujikawa K, Sasaki M, Aoyama T and Itoh T (1995*b*) Prognostic criteria in patients with stage D2 prostate cancer. *Cancer* **76**: 91–95

Gundersen HJG and Jensen EB (1985) Stereological estimation of the volume-weighted mean volume of arbitrary particles observed on random sections. *J Microsc* **138**: 127–142

Jorgensen PE, Berild GH, Bruun E, Weis N, Nielsen K and Gundersen JG (1988) Stereological estimation of nuclear volume correlated with clinical stage and progression of prostatic cancer: clinical aspects. *Scand J Urol Suppl* **110**: 131–135

Nielsen K, Colstrup H, Nilsson T and Gundersen HJG (1986) Stereological estimates of nuclear volume correlated with histopathological grading and prognosis of bladder tumor. *Virchows Arch* **52**: 41–54

Nielsen K, Petersen SE and Omtoft T (1989*a*) A comparison between stereological estimates of mean nuclear volume and DNA flow cytometry in bladder tumours. *APMIS* **97**: 949–956

Nielsen K, Berild GH, Bruun E, Jorgensen PE and Weis N (1989*b*) Stereological estimation of mean nuclear volume in prostatic cancer, the reproducibility and the possible value of estimations on repeated biopsies in the course of disease. *J Microsc* **154**: 63–69

Partin AW, Steinberg GD, Pitcock RV, Wu L, Piantadosi S, Coffey D and Epstein JI (1992) Use of nuclear morphometry, Gleason histological scoring, clinical stage, and age to predict disease-free survival among patients with prostatic cancer. *Cancer* **70**: 161–168

Song J, Cheng WS, Cupps RE, Earle JD and Lieber MM (1992) Nucleardeoxyribonucleic acid content measured by static cytömetry: important prognostic association for patients with clinically localized prostate carcinoma treated by external beam radiotherapy. *J Urol* **147**: 794–797

Svanholm H and Myging H (1985) Prostatic carcinoma reproducibility of histologic grading. *Acta Path Microbiol Immunol Scand* **93**: 67–71

© Cancer Research Campaign 1997